# Retraction: Commentary: One mechanism of Sishen Pill on diarrhea with kidney Yang deficiency syndrome: influencing metabolic function by intestinal microorganisms and enzyme activity mediates the gut-kidney axis

**DOI:** 10.3389/fcimb.2026.1900127

**Published:** 2026-06-18

**Authors:** Frontiers Editorial Office

The Journal retracts the 15 September 2025 article cited above.

Following publication, concerns were raised that the handling Editor was an author on the original article that the Commentary is discussing. Our investigation, conducted in accordance with Frontiers policies, confirmed an undisclosed conflict of interest that undermined the integrity of the peer review process. As the scientific integrity of the article cannot be guaranteed, and in adherence to the recommendations of the Committee on Publication Ethics (COPE), the article is retracted.

This retraction was approved by the Chief Executive Editor of Frontiers. The authors have not responded to correspondence regarding this retraction.

